# MiR‐22‐3p inhibits fibrotic cataract through inactivation of HDAC6 and increase of α‐tubulin acetylation

**DOI:** 10.1111/cpr.12911

**Published:** 2020-09-28

**Authors:** Xiaoran Wang, Liping Wang, Yan Sun, Baoxin Chen, Lang Xiong, Jieping Chen, Mi Huang, Jing Wu, Xuhua Tan, Yingfeng Zheng, Shan Huang, Yizhi Liu

**Affiliations:** ^1^ State Key Laboratory of Ophthalmology Zhongshan Ophthalmic Center Sun Yat‐sen University Guangzhou China

## Abstract

**Objectives:**

Fibrotic cataract, including posterior capsule opacification (PCO) and anterior subcapsular cataract (ASC), renders millions of people visually impaired worldwide. However, the underlying mechanism remains poorly understood. Here, we report a miRNA‐based regulatory pathway that controls pathological fibrosis of lens epithelium.

**Materials and methods:**

Expression of miR‐22‐3p and histone deacetylase 6 (HDAC6) in normal and PCO patient samples were measured by qPCR. Human lens epithelial explants were treated with TGF‐β2 in the presence or absence of miR‐22‐3p mimics or inhibitor. Cell proliferation was determined by MTS assay, and migration was tested by transwell assay. Expression of HDAC6 and EMT‐related molecules were analysed by Western blot, qPCR and immunocytochemical experiments.

**Results:**

We identify miR‐22‐3p as a downregulated miRNA targeting HDAC6 in LECs during lens fibrosis and TGF‐β2 treatment. Mechanistically, gain‐ and loss‐of‐function experiments in human LECs and lens epithelial explants reveal that miR‐22‐3p prevents proliferation, migration and TGF‐β2 induced EMT of LECs via targeting HDAC6 and thereby promoting α‐tubulin acetylation. Moreover, pharmacological targeting of HDAC6 deacetylase with Tubacin prevents fibrotic opaque formation through increasing α‐tubulin acetylation under TGF‐β2 stimulated conditions in both human lens epithelial explants and the whole rat lenses.

**Conclusions:**

These findings suggest that miR‐22‐3p prevents lens fibrotic progression by targeting HDAC6 thereby promoting α‐tubulin acetylation. The ‘miR‐22‐HDAC6‐α‐tubulin (de)acetylation’ signalling axis may be therapeutic targets for the treatment of fibrotic cataract.

## Graphical abstract

Schematic diagram depicting the role of ‘miR‐22‐HDAC6‐α‐tubulin (de)acetylation’ signalling axis in fibrotic cataract. miR‐22‐3p, which represses HDAC6 expression post‐transcriptionally and thereby promotes α‐tubulin acetylation, is downregulated during lens fibrosis. The ‘miR‐22‐HDAC6‐α‐tubulin (de)acetylation’ signalling axis regulates LECs proliferation, migration and EMT, the fundamental pathogenic processes trigging fibrotic cataract.

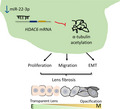

## 1 INTRODUCTION

The crystalline lens, composed of only mono‐layered lens epithelium and orderly arranged lens fibres with a basement membrane surrounded, is a remarkable structure that contributes to focusing of images on the retina. Opacification in lens, the cataract, is the leading cause of blindness worldwide.^1^ Surgery is currently the only effective treatment available. However, the most common complication, known as posterior capsule opacification (PCO), could result in visual axis opacification once again due to the migration of the remaining lens epithelial cells (LECs) towards the posterior lens capsule with excessive proliferation and mesenchymal transformation in response to the surgical injury.^2^ In paediatric cataract patients, particularly, the incidence of PCO is almost 100%, which could disrupt their visual development and may result in irreversible blindness.^3^ Apart from PCO, fibrotic opacities could also accumulate underneath the anterior capsular induced by ocular trauma, inflammation or radiation, namely anterior subcapsular cataract (ASC).^4^

In fibrotic cataracts, lens epithelium loss their integrity and function while disorganized mesenchymal‐like cells accumulate with excessive fibronectin (FN) and collagen (Col) secreted into the ECM, thereby damaging the architecture of the lens and leading to eventual visual loss.^5^ TGF‐βs are considered as the most prominent pro‐fibrotic factors, and TGF‐β2 is the major isoform in the aqueous humour.^6^ Therefore, deciphering the underlying mechanism of proliferation, migration and TGF‐β2‐induced EMT in lens is of great value for the prevention and treatment of fibrotic cataract.

MicroRNAs (miRNAs), a group of small non‐coding RNA strands that regulate their targets via translational repression or mRNA degradation, are involved in diverse biological cellular processes.^7^ MiRNAs level is controlled by the TGF‐β signalling pathway in both direct and indirect manners in fibrotic diseases. SMADs, the key TGF‐β signal transducer, could directly regulate miRNAs expression through several mechanisms, including the inhibition of pri‐miRNA transcription, Drosha‐mediated pri‐miRNA processing and the maturation of miRNAs.^8^, ^9^ Moreover, TGF‐β could indirectly affect miRNAs, such as miR‐203 and miR‐200 family, through downstream transcriptional factors, like ZEB1, ZEB2, SNAIL and SLUG.^10^, ^11^ Increasing evidence has demonstrated that dysregulation of miRNAs, such as let‐7a‐5p, miR‐34a, miR‐181a and miR‐204‐5p,^12^, ^13^, ^14^, ^15^ is associated with EMT process during cataract. However, the specific effect of miR‐22‐3p in lens fibrosis remains unexplored. In this study, miR‐22‐3p was found to decrease significantly in both fibrotic cataract tissue and TGF‐β2 induced fibrosis model of human lens epithelial explants, implying miR‐22‐3p as a repressor during the pathogenesis of fibrotic cataracts.

Our previous study has identified HDAC6 as a target gene of miR‐22‐3p in mesenchymal stem cells.^16^ Histone deacetylases (HDACs) are a group of enzymes that control chromatin condensing and gene transcription through deacetylation on lysine residues mainly in histones.^17^, ^18^ Studies have shown that HDAC inhibition prevents EMT of LECs and suggested the epigenetic modifiers as potential targets to control lens fibrosis.^19^, ^20^ Distinguished from other HDAC members, HDAC6 is a primary cytoplasmic deacetylase owing to its association with microtubules and could deacetylase cytoplasmic substrates besides chromatin histones to initiate signalling events, including embryonic development, tumorigenesis and neurodegenerative diseases.^21^, ^22^, ^23^ Emerging evidences reveal HDAC6 as a critical regulator in renal,^24^ peritoneal^25^ and pulmonary fibrosis^26^; however, the effect of HDAC6 on ocular fibrosis remains unknown.

In this study, we identify miR‐22‐3p as a key regulator that prevents lens fibrosis. We show that miR‐22‐3p decreases significantly in PCO cataract and that TGF‐β2 markedly downregulates miR‐22‐3p expression in fibrotic human lens epithelial explants. Moreover, HDAC6, a direct target of miR‐22‐3p, is increased and activated in these settings and controls lens fibrosis. Importantly, we reveal a novel mechanism underlying TGF‐β2‐induced lens fibrosis by showing the effect of miR‐22‐3p downregulation on proliferation, migration and EMT of LECs, via increasing HDAC6 and consequently decreasing α‐tubulin acetylation. Furthermore, using of HDAC6 deacetylase inhibitor ‘Tubacin’ can reverse ASC development. Our study identifies a regulatory pathway in LECs consisting of miR‐22‐3p, HDAC6 and α‐tubulin (de)acetylation that controls lens fibrosis. Modulation of this pathway may provide a therapeutic option for fibrotic cataracts.

## 2 MATERIALS AND METHODS

### 2.1 Lens epithelial explants collection, cell culture and treatment

All human samples were gathered from organ donors provided by the Eye Bank of Zhongshan Ophthalmic Center (Guangdong, China) in accordance with the Declaration of Helsinki. Totally 29 fresh post‐mortem human lenses from donors (ages ranged from 40 to 55 years, 23 males and 6 females) collected within 8 hours after death were used in our study. Three of the lenses were PCO tissues from donors who had cataract surgery before death, and the fibrotic part of the posterior capsule was isolated with the capsulotomy vannas scissors. For the 26 normal transparent lenses, anterior capsules with attached epithelium were dissected with capsulotomy scissors along the lens equator region and stripped away from the lens fibre by capsulorhexis forceps for lens epithelial explants collection. Lens epithelial explants were cultured with the epithelium side up in the Minimum Essential Medium (MEM, Thermo Fisher Scientific) containing 1% foetal bovine serum (FBS, Thermo Fisher Scientific) with 1% NEAA (Life Technologies), 100 IU/mL penicillin and 100 µg/mL streptomycin (Life Technologies). For the TGF‐β2‐induced lens fibrosis model, explants were treated with 5 ng/mL TGF‐β2 (302‐B2; R&D) for 48‐72 hours. The human lens epithelial cell line SRA 01/04 was kindly provided by Professor Fu Shang of Tufts University (Boston, MA, USA) and cultured in Dulbecco's modified Eagle's medium (DMEM, Thermo Fisher Scientific) with 10% FBS.

### 2.2 In vitro transfection with miRNA mimics, miRNA inhibitors and siRNA

The synthetic miR‐22‐3p mimics, inhibitors and interfering RNAs of HDAC6 (siHDAC6) were from Life Technologies. About 50 nmol/L miRNA mimics, inhibitors or 40 nmol/L siHDAC6 was transfected to human lens epithelial explants or human LECs for gain‐ and loss‐of‐function studies by using siPORT™ NeoFX™ Transfection Agent (AM‐4511; Applied Biosystems) according to the manufacturer's instructions. The detailed sequences are listed in Table S1.

### 2.3 Whole‐mount staining with immunofluorescence staining analysis

The whole‐mount lens epithelial explants were flattened on a glass slide and fixed with 4% paraformaldehyde for 20 minutes after TGF‐β2 treatment and/or transfection. The samples were blocked in PBS solution containing 0.3% Triton X‐100 and 3% BSA for 30 minutes, followed by overnight incubation with primary antibodies (1:100) at 4°C. Then, the samples were incubated with secondary antibodies for 1 hour and counterstained with DAPI (Sigma‐Aldrich). The following antibodies were used: rabbit anti‐fibronectin polyclonal antibody (ab137720; Abcam), mouse anti‐α‐SMA monoclonal antibody (ab7817; Abcam), rabbit anti‐Ki67 polyclonal antibody (AB9260; Millipore), rabbit anti‐ZO‐1 polyclonal antibody (61‐7300; Thermo Fisher Scientific), rabbit anti‐β‐catenin monoclonal antibody (8480; CST), rabbit anti‐HDAC6 polyclonal antibody (07‐732; Millipore) and rabbit‐acetylated‐α‐tubulin monoclonal antibody (5335; CST). Images were obtained using a Leica DM3000 microscope system.

### 2.4 RNA isolation and qPCR

Total RNA from fibrotic PCO tissues or lens epithelial explants were purified using Arcturus PicoPure RNA isolation kit (KIT0204; Applied Biosystems) according to the manufacturer's protocol. cDNAs of miRNAs were synthesized by miScript II RT Kit (218160; Qiagen), and qPCR analysis of miR‐22‐3p was performed using miScript SYBR Green PCR Kit (218073; Qiagen). cDNAs of mRNAs were synthesized with Maxima First strand cDNA synthesis kit (K1641; Thermo Fisher Scientific) and qPCR was performed using FastStart Universal SYBR Green Master (Rox) (4913914001‐1; Roche). The amplification was performed on a StepOnePlus real‐time PCR system (Applied Biosystems) on standard settings. The expression levels were normalized to that of U6 or GAPDH, respectively. The sequences of oligonucleotide primers used in this study are listed in Table S1.

### 2.5 Western blot analysis

Lens epithelial explants were lysed with radioimmunoprecipitation assay buffer supplemented with protease inhibitors (P0013C; Beyotime). Proteins were separated with SDS‐PAGE and transferred onto PVDF membranes. The membranes were saturated with 5% non‐fat milk and incubated overnight at 4°C with the following primary antibodies (1:1000): mouse anti‐β‐actin monoclonal antibody (3700; CST), rabbit anti‐HDAC6 polyclonal antibody (07‐732; Millipore), rabbit‐acetylated‐α‐tubulin monoclonal antibody (5335; CST) and rabbit α‐tubulin monoclonal antibody (2125; CST). Secondary HRP‐conjugated antibodies (CST) were added for 2 hours. Finally, the bands of the membranes were revealed with the chemiluminescence detection systems (Protein simple).

### 2.6 Proliferation assay

We used MTS Cell Proliferation Colorimetric Assay Kit (G3580; Promega) to determine the proliferation abilities of cells as instructions. The cells were seeded into 96‐well plates at a density of 2 × 10^4^ cells/ well. MTS reagent was added and incubated at 37°C for 1 hour from day 0 to day 5, and the absorbance values of each well were measured by using a spectrophotometer at 490 nm.

### 2.7 Transwell migration assay

Human LECs (2 × 10^5^) were dispensed into the upper wells of the transwell chamber with 8‐µm pores (Corning), while the lower wells contained medium with 10% FBS as the chemoattractant. After incubation for 24 hours, the cells on the upper surfaces of the filter were swabbed and those on the lower surfaces were fixed with 4% paraformaldehyde for 30 minutes. The migrated cells were dyed with crystal violet (Beyotime) and counted with microscope (Zeiss AxioObserver A1).

### 2.8 Detection of HDAC6 deacetylase activity

The activity of HDAC6 was determined using the HDAC6 Activity Assay Kit (K466‐100; Bio Vision) according to the manufacturer's protocol. Briefly, extract (2 μg total protein) was incubated at 37°C with 2 μL HDAC6 Substrate in HDAC6 Assay Buffer to 100 μL for 30 minutes. The reaction was stopped by adding 10 μL Developer to each well at 37°C for 10 minutes. Fluorescence was measured by using a Synergy H1 Hybrid Reader (BioTek) with excitation at 380 nm and emission at 490 nm. HDAC6 activity was calculated by Relative Fluorescence Unites per milligram of the sample.

### 2.9 Model of TGF‐β2‐induced ASC in whole lens culture

All animal procedures were carried out in accordance with the ARVO Statement for the Use of Animals in Ophthalmic and Vision Research and were approved by the Animal Use and Care Committee of Zhongshan Ophthalmic Center at Sun Yat‐Sen University. Whole lenses of 21‐day‐old Wistar rats were cultured in 4 mL serum‐free M199 medium with 0.1% BSA, 0.1 µg/mL l‐glutamine, 100 IU/mL penicillin and 100 µg/mL streptomycin. Different concentrations of Tubacin (SML0065; Sigma‐Aldrich) were added to the medium with or without 5 ng/mL TGF‐β2 for up to five days. On Day 5, all lenses were fixed with 10% formaldehyde and embedded in paraffin for further immunofluorescence analysis.

### 2.10 Statistical analysis

Comparisons between two groups were examined using Student's *t* test while the differences among multiple groups were analysed using One‐way ANOVA. A value of *P* < .05 was considered statistically significant.

## 3 RESULTS

### 3.1 MiR‐22‐3p is downregulated in PCO tissues and explants

**Figure 1:**
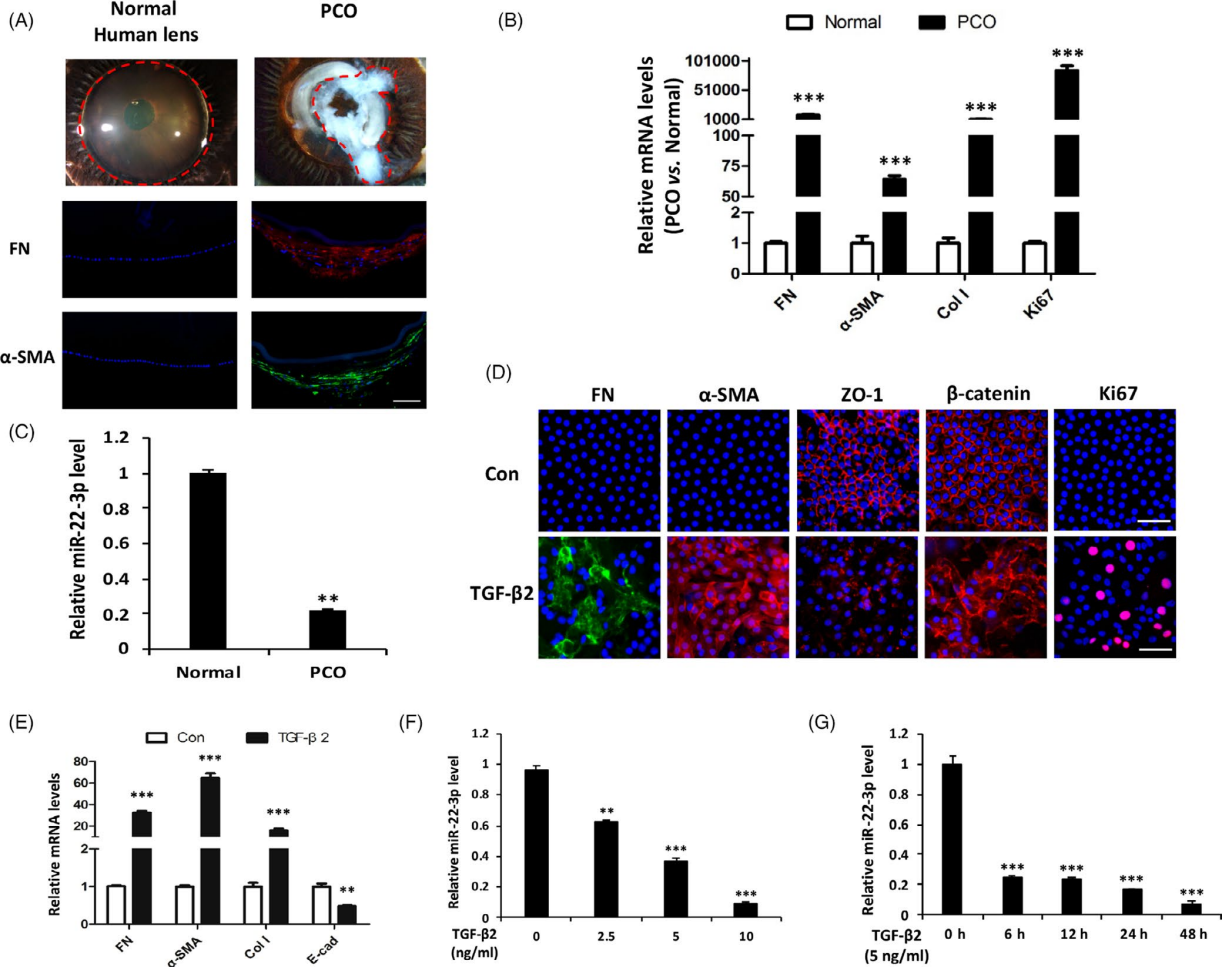
MiR‐22‐3p is downregulated in PCO tissues and explants. A, Transparent normal lens and fibrotic PCO tissues from donors. FN (red) and α‐SMA (green) merged with DAPI (blue) in normal lens and PCO tissues by immunofluorescence staining. scale bars: 100 μm. B, Comparison of gene expression levels (FN, α‐SMA, Col I and Ki67) in normal lens epithelium vs PCO tissues by qPCR analysis. C, qPCR analysis of miR‐22‐3p in PCO tissues relative to normal lens epithelium. D, Immunofluorescence staining of FN, α‐SMA, ZO‐1, β‐catenin and Ki67 in lens epithelial explants treated with or without TGF‐β2 (5 ng/mL) for 48 h. scale bars: 50 μm. E, Comparison of gene expression levels (FN, α‐SMA, Col I and E‐cad) in lens epithelium treated with or without TGF‐β2 (5 ng/mL) for 48 h by qPCR analysis. F and G, qPCR analysis of the expression of miR‐22‐3p in response to TGF‐β2 in lens epithelial explants with different concentrations (2.5, 5 and 10 ng/mL) for 6 h (F) and at different time points (6, 12, 24 and 48 h) (G). **P < .01 and ***P < .001 by Student's *t* test, all n = 3 per group. Data are shown as mean ± SD

Transparent lens capsules and fibrotic lens capsules from healthy donors and PCO patients were collected. Expression levels of mesenchymal marker α‐SMA, ECM markers FN and Col I as well as proliferative marker Ki67 were markedly increased in PCO tissues relative to normal lens epithelium (Figure 1A,B), which indicates that lens fibrosis involves a combination of processes including hyperproliferation and EMT. Interestingly, miR‐22‐3p was significantly downregulated in PCO tissues than that of normal tissues (Figure 1C). To further explore the regulatory role of miR‐22‐3p in lens fibrosis, human lens explants were stimulated with TGF‐β2, the main type of pro‐fibrotic cytokines in aqueous humour. As shown in Figure 1D,E, lens epithelial explants treated with TGF‐β2 lost epithelial features with reduced Zonula occludens‐1 (ZO1) and E‐cadherin (E‐cad) expression as well as β‐catenin translocated from cell membrane to cytoplasm, and transformed into mesenchymal‐like cells indicated by increases of α‐SMA, FN and Col I, with both morphological and molecular changes resembling the fibrotic cataract. Notably, miR‐22‐3p decreased significantly upon TGF‐β2 stimulation in both dose‐ and time‐dependent manners (Figure 1F,G). These findings suggest miR‐22‐3p is downregulated in PCO tissues and explants, which may be correlated with lens fibrosis development.

### 3.2 MiR‐22‐3p prevents proliferation, migration and TGF‐β2‐dependent EMT of LECs

**Figure 2:**
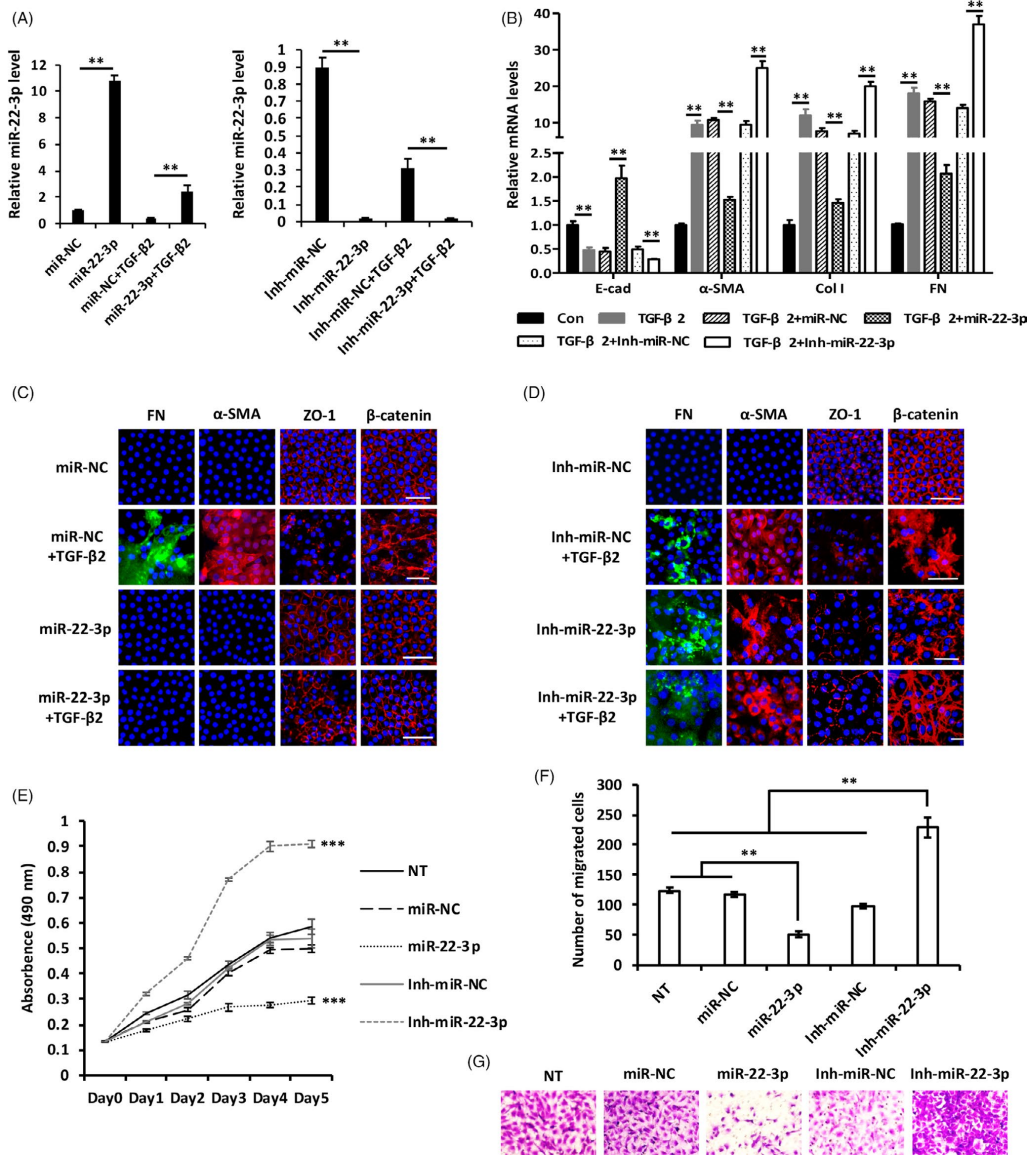
MiR‐22‐3p prevents proliferation, migration and TGF‐β2‐dependent EMT of LECs. A, MiR‐22‐3p levels in lens epithelial explants by qPCR after being transfected with miR‐22‐3p mimics or inhibitors for 24 h and followed by further treatment with or without 5 ng/mL TGF‐β2 for 24 h. B‐D, Changes of EMT‐related markers after transfected with mimics and inhibitors of miR‐22‐3p in TGF‐β2 induced fibrosis model of lens epithelial explants detected by qPCR (B) and immunofluorescent staining (C, D). E, Proliferative activity of LECs after transfected with mimics and inhibitors of miR‐22‐3p. F and G, Quantification of lower chambers (F) and representative images (G) following Transwell migration assays after overexpression or inhibition of miR‐22‐3p. All scale bars: 50 μm. **P < .01 and ***P < .001 by Student's *t* test, all n = 3 per group. Data are shown as mean ± SD

To explore the functional impact of miR‐22‐3p on lens fibrosis, we performed gain‐ and loss‐of‐function studies by transfecting miR‐22‐3p mimics or inhibitor into human lens epithelial explants treated with TGF‐β2 (Figure 2A). Surprisingly, ectopic expression of miR‐22‐3p largely blocked TGF‐β2‐induced EMT of LECs by preventing mesenchymal‐transformed markers of α‐SMA, FN and Col I while retaining epithelial markers of ZO1 and E‐cad upon TGF‐β2 treatment (Figure 2B,C). Meanwhile, overexpression of miR‐22‐3p restored the epithelial morphology and prevented β‐catenin translocating from the cell membrane into the cytoplasm following TGF‐β2 incubation (Figure 2C). Consistently, miR‐22‐3p inhibitors promoted EMT of lens epithelial explants (Figure 2B,D). Moreover, miR‐22‐3p overexpression in human lens SRA01/04 cells inhibited while its downregulation promoted proliferation and migration of LECs compared with the miR‐NC‐ or Inh‐NC‐transfected group (Figure 2E‐G).

### 3.3 MiR‐22‐3p inhibits LECs EMT by regulating HDAC6 expression post‐transcriptionally

**Figure 3:**
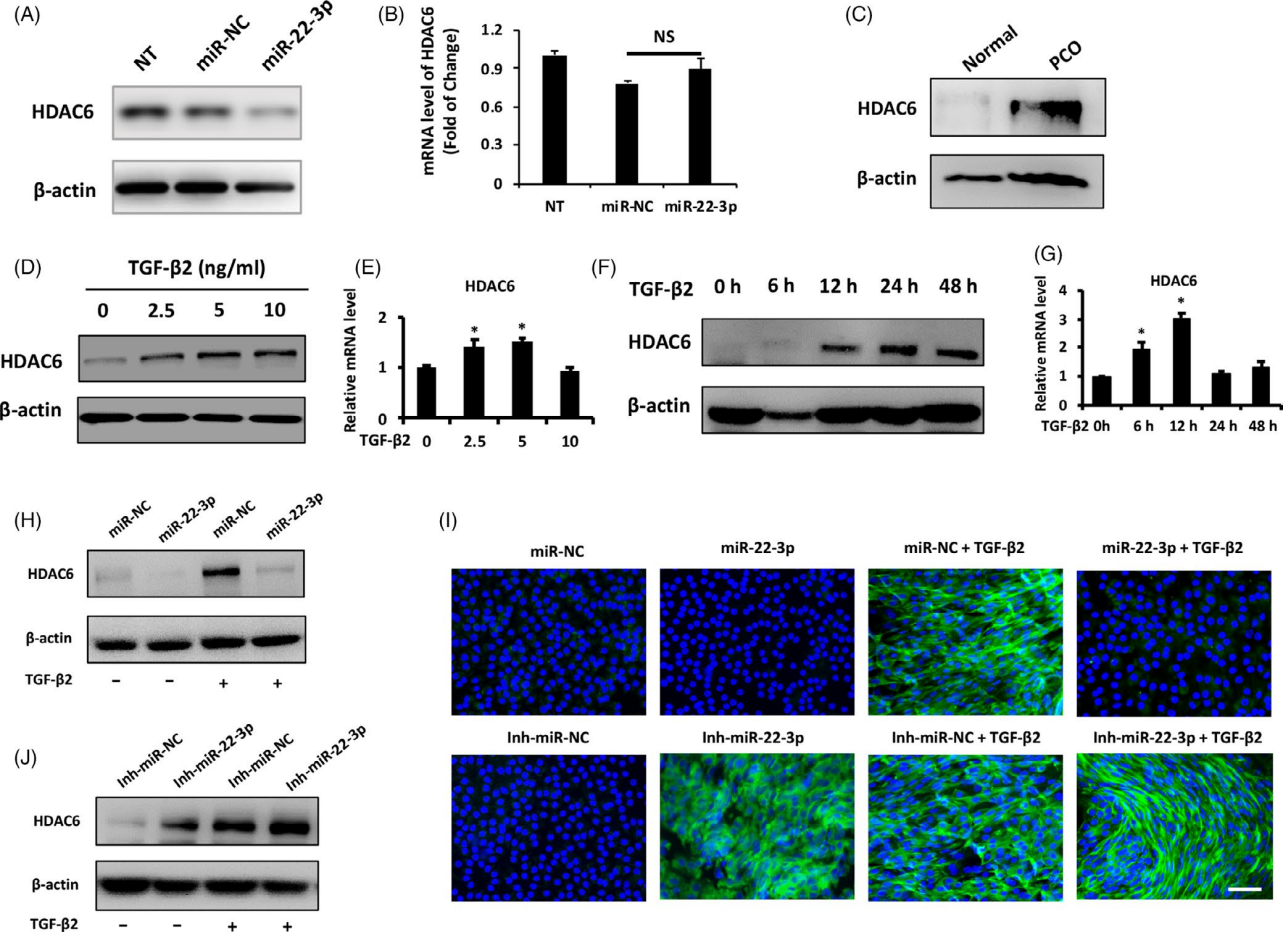
MiR‐22‐3p inhibits LECs EMT by regulating HDAC6 expression post‐transcriptionally. A, Western blot analysis of HDAC6 protein level in non‐transfected (NT), miRNA‐negative control (miR‐NC) and miR‐22‐3p mimic‐transfected lens epithelial explants after 48 h transfection. B, qPCR analysis of HDAC6 mRNA level in NT, miR‐NC and miR‐22‐3p mimic‐transfected lens epithelial explants after 48 h transfection. C, Western blot analysis of HDAC6 protein level in the normal lens epithelium and PCO tissues. D, Western blot and qPCR analysis (E) of HDAC6 expression levels after treated by TGF‐β2 with different concentrations (2.5, 5 and 10 ng/mL) for 6 h. F, Western blot and qPCR analysis (G) of HDAC6 expression levels in response to TGF‐β2 (5 ng/mL) treatment at different time points (6, 12, 24 and 48 h), untreated lens epithelial explant was considered as controls. Western blot (H, I) and immunofluorescence staining (J) of HDAC6 expression levels after transfection with miR‐22‐3p mimics or inhibitors for 24 h and further treatment with or without TGF‐β2 (5 ng/mL) for 24 h in lens epithelial explants. All scale bars: 50 μmol/L. NS, not significant. *P < .05 by Student's *t* test, all n = 3 per group. Data are shown as mean ± SD

Our previous study has shown that miR‐22‐3p targets the 3’UTR of HDAC6 mRNA in mesenchymal stem cells.^16^ After transfecting LECs with miR‐22‐3p mimics, we found that increasing miR‐22‐3p repressed HDAC6 protein expression by 88% in LECs (Figure 3A) without affecting its mRNA level (Figure 3B), demonstrating that HDAC6 is negatively regulated by miR‐22‐3p in a post‐transcriptional manner in the lens. To confirm the potential role of HDAC6 during lens fibrosis, we analysed HDAC6 expression levels in PCO tissues and normal lens epithelium. Notably, HDAC6 dramatically increased in PCO tissues compared to normal LECs (Figure 3C). Expressions of HDAC6, particularly in protein levels, were upregulated significantly by TGF‐β2 in lens epithelial explants in both dose‐ (Figure 3D,E) and time‐dependent manners (Figure 3F,G).

Furthermore, miR‐22‐3p mimics significantly downregulated HDAC6 levels in lens epithelial explants while miR‐22‐3p inhibitors upregulated HDAC6 expression levels with or without TGF‐β2 (Figure 3H‐J), indicating that HDAC6 is a direct target of miR‐22‐3p during lens fibrosis.

### 3.4 HDAC6 inhibition suppresses proliferation, migration and TGF‐β2‐dependent EMT

**Figure 4:**
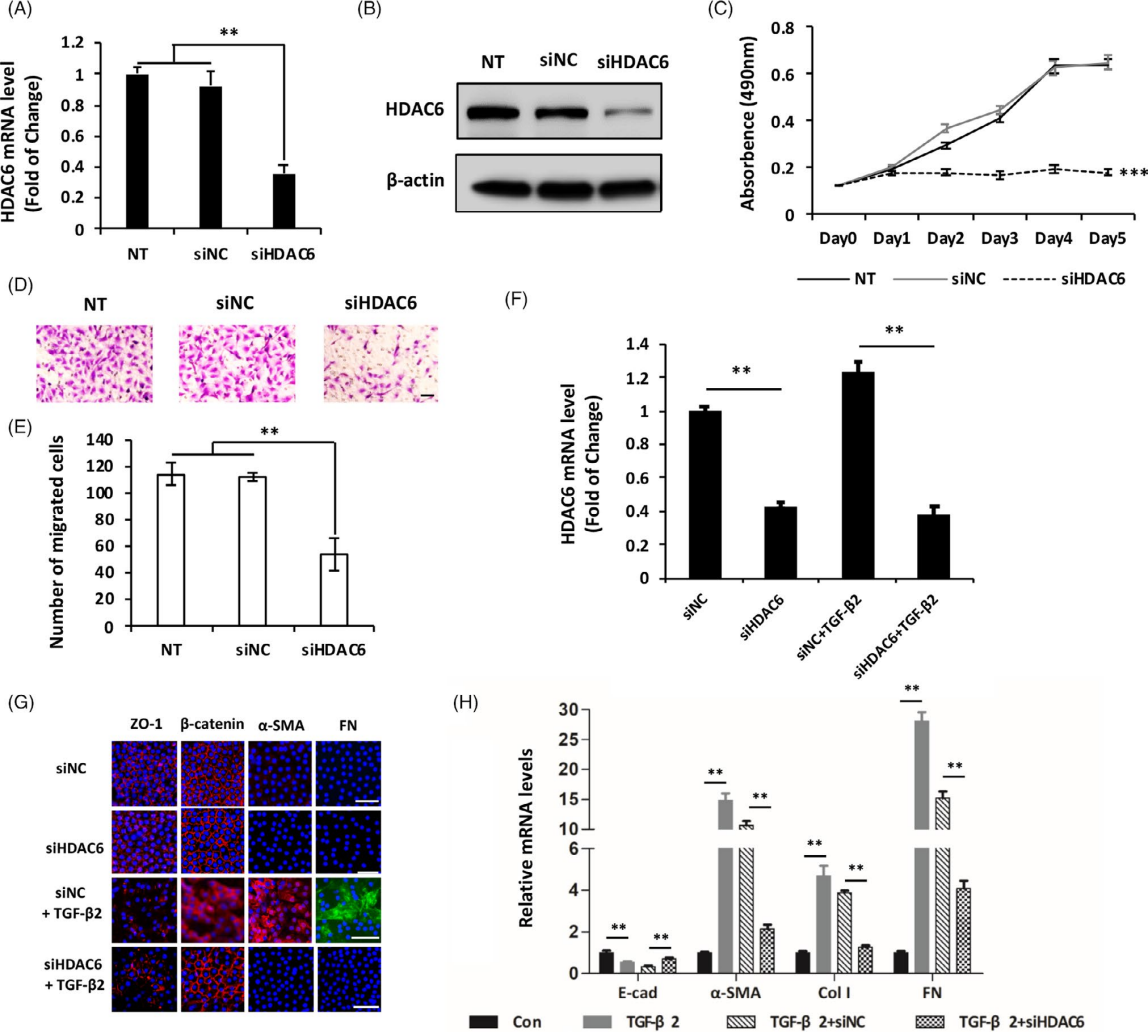
HDAC6 inhibition suppresses proliferation, migration and TGF‐β2‐dependent EMT. A and B, qPCR and Western blot analysis of HDAC6 expression levels in non‐transfected (NT), siRNA negative control (siNC) and siRNA of HDAC6 (siHDAC6)‐transfected human LECs after 48 h transfection. C, Proliferative activity of LECs after transfected with siHDAC6 and siNC. D and E, Representative images (D) and quantification (E) of lower chambers following Transwell migration assays after HDAC6 knockdown by siRNA. F, HDAC6 mRNA level in lens epithelial explants by qPCR after transfected with siHDAC6 for 12 h and further treated with or without TGF‐β2 (5 ng/mL) for 24 h. G and H, Changes of EMT‐related markers after transfected with siHDAC6 in TGF‐β2 induced fibrosis model of lens epithelial explants detected by immunofluorescent staining (G) and qPCR (H). All scale bars: 50 μm. **P < .01 and ***P < .001 by Student's *t* test, all n = 3 per group. Data are shown as mean ± SD

To investigate whether HDAC6 mediate the biological functions of miR‐22‐3p during lens fibrosis, we conducted a loss‐of‐function approach by knocking down HDAC6 in LECs. Introduction of a siRNA targeting HDAC6 mRNA (siHDAC6) effectively inhibited the expression of HDAC6 in SRA01/04 cells (Figure 4A,B) and blocked proliferation (Figure 4C) and migration of LECs (Figure 4D,E). Moreover, lens epithelial explants treated by TGF‐β2 exhibited restoration of epithelial state and diminished mesenchymal markers with HDAC6 knockdown (Figure 4F‐H). Collectively, these results demonstrate that HDAC6 is required for LECs transforming into mesenchymal‐like cells and strengthen the finding that miR‐22‐3p controls lens fibrosis by targeting HDAC6.

### 3.5 Involvement of miR‐22‐3p‐HDAC6‐α‐tubulin (de)acetylation axis in lens fibrosis

HDAC6 can shuttle between cytoplasm and nucleus to deacetylase both non‐histone and histone substrates under certain contexts.^23^ In this study, HDAC6 was shown to accumulate exclusively in the cytoplasm of the elongated and spindle‐shaped mesenchymal cells upon TGF‐β2‐induced fibrosis of lens (Figure 6B), excluding the possibility of its modification on nuclear histones and emphasizing its cytoplasmic deacetylation effect during lens fibrosis. Microtubule α‐tubulin, the primary component of the cytoskeleton, represents the most important substrate of HDAC6.^27^ It is worth noting that cytoskeleton, the basic cellular structure, affects nearly all the cellular processes^28^ and that EMT is a process by which epithelial cells reorganize their cytoskeleton to obtain mesenchymal morphology.^29^ However, the regulatory network and the role of α‐tubulin acetylation on lens fibrosis have not been explored.

**Figure 5:**
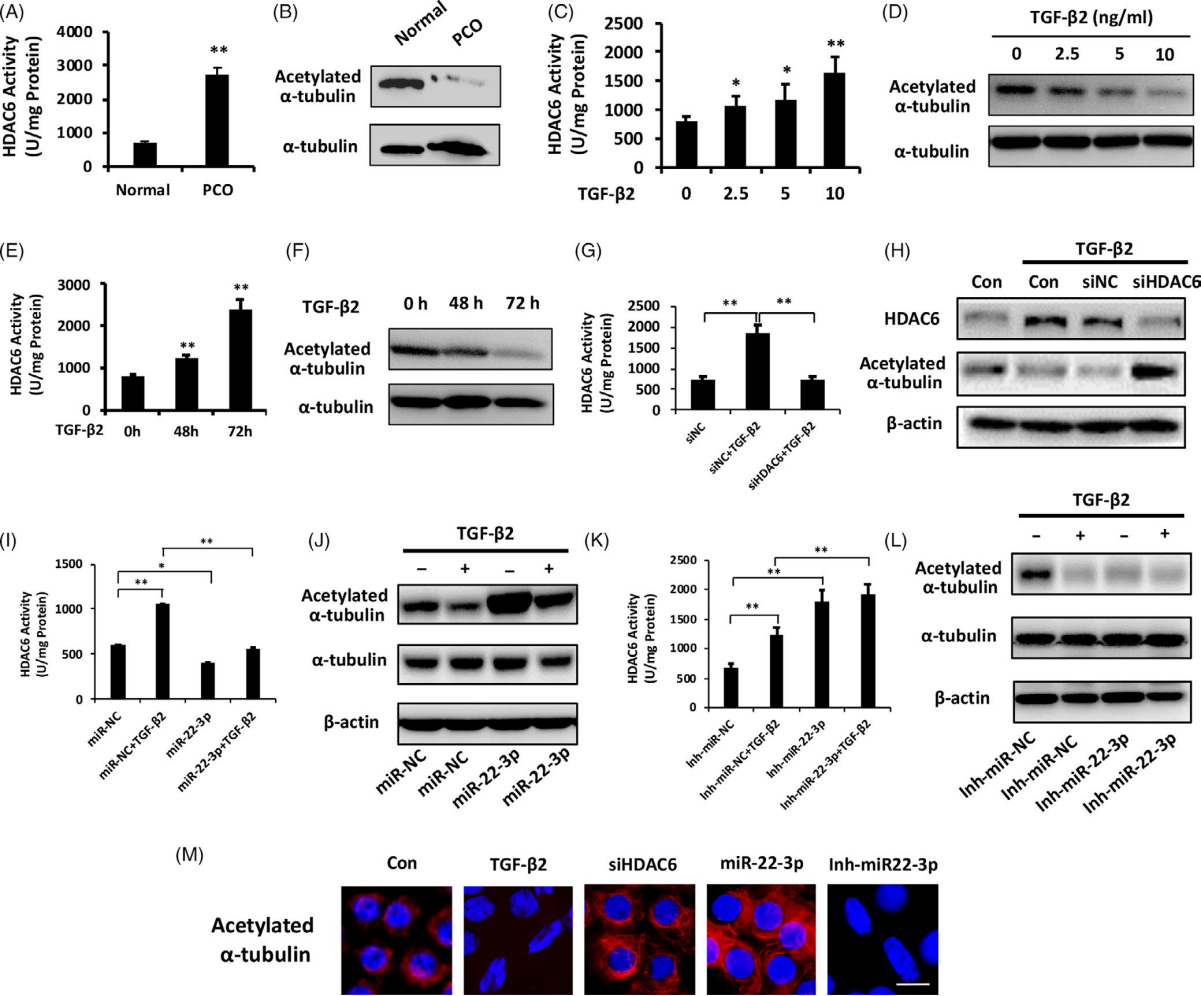
Involvement of miR‐22‐3p‐HDAC6‐α‐tubulin (de)acetylation axis in lens fibrosis. A, Comparison of HDAC6 deacetylase activity in normal lens epithelium and PCO tissues. B, Western blot analysis of acetylated α‐tubulin in PCO tissues vs normal lens epithelium. C and D, Activity of HDAC6 deacetylase (C) and acetylated α‐tubulin levels (D) in lens epithelial explants in response to TGF‐β2 with different concentrations (2.5, 5 and 10 ng/mL). E and F, Activity of HDAC6 deacetylase (E) and acetylated α‐tubulin levels (F) in lens epithelial explants in response to TGF‐β2 (5 ng/mL) at different time points (48 and 72 h). G, HDAC6 deacetylase activity in lens epithelium after 12 h transfection by siHDAC6 or siNC and further treatment with or without TGF‐β2 for 24 h. H, Western blot analysis of acetylated α‐tubulin in lens epithelium after HDAC6 knockdown under TGF‐β2 treated conditions. I and J, Effect of miR‐22‐3p overexpression on HDAC6 deacetylase activity (I) and acetylated α‐tubulin levels (J) in lens with or without TGF‐β2 treatment for 24 h. K and L, Effect of miR‐22‐3p inhibitor on HDAC6 deacetylase activity (K) and acetylated α‐tubulin levels (L) in lens with or without TGF‐β2 treatment for 24 h. M, α‐tubulin acetylation by immunofluorescence staining analysis after TGF‐β2 treatment, siHDAC6, miR‐22‐3p mimics and miR‐22‐3p inhibitor transfection in lens epithelial explants. Scale bar: 10 μm. *P < .05, **P < .01 by Student's *t* test, all n = 3 per group. Data are shown as mean ± SD

To clarify the underlying mechanism of HDAC6 on lens fibrosis, we detected the HDAC6 deacetylase activity and acetylated α‐tubulin levels in the PCO tissues and TGF‐β2‐induced fibrotic lens. Deacetylase activity of HDAC6 revealed a nearly 4‐fold increase in PCO tissues compared with normal lens epithelium (Figure 5A). In contrast, acetylated‐α‐tubulin was decreased by 91.2% in PCO tissues relative to normal lens epithelium (Figure 5B). Moreover, HDAC6 deacetylase activity was elevated notably while α‐tubulin acetylation was inhibited significantly by TGF‐β2 in dose‐ and time‐dependent manners (Figure 5C‐F). These data reveal a significant suppression of α‐tubulin acetylation with increased HDAC6 deacetylase activity during lens fibrosis.

To determine the association between miR‐22‐3p and α‐tubulin (de)acetylation in LECs induced by TGF‐β2, we evaluated whether HDAC6 could trigger α‐tubulin deacetylation under TGF‐β2‐treated conditions. HDAC6 knockdown prevented elevation of its deacetylase activity induced by TGF‐β2 (Figure 5G) and thereby promoted α‐tubulin acetylation (Figure 5H). Furthermore, miR‐22‐3p mimics significantly prevented TGF‐β2‐induced HDAC6 activity elevation (Figure 5I) while promoted α‐tubulin acetylation (Figure 5J) and maintained epithelial morphology of LECs (Figure 5M). In contrast, miR‐22‐3p inhibitors markedly enhanced HDAC6 deacetylase activity (Figure 5K) and decreased acetylated α‐tubulin levels with or without TGF‐β2 (Figure 5L). Interestingly, inhibition of miR‐22‐3p in LECs showed spindle‐shaped cells morphological transformation with α‐tubulin deacetylation even without TGF‐β2 (Figure 5M). These results imply ‘miR‐22‐3p‐HDAC6‐α‐tubulin (de)acetylation’ as a regulatory pathway during EMT.

### 3.6 Inhibition of HDAC6 prevents lens fibrosis through α‐tubulin acetylation

**Figure 6:**
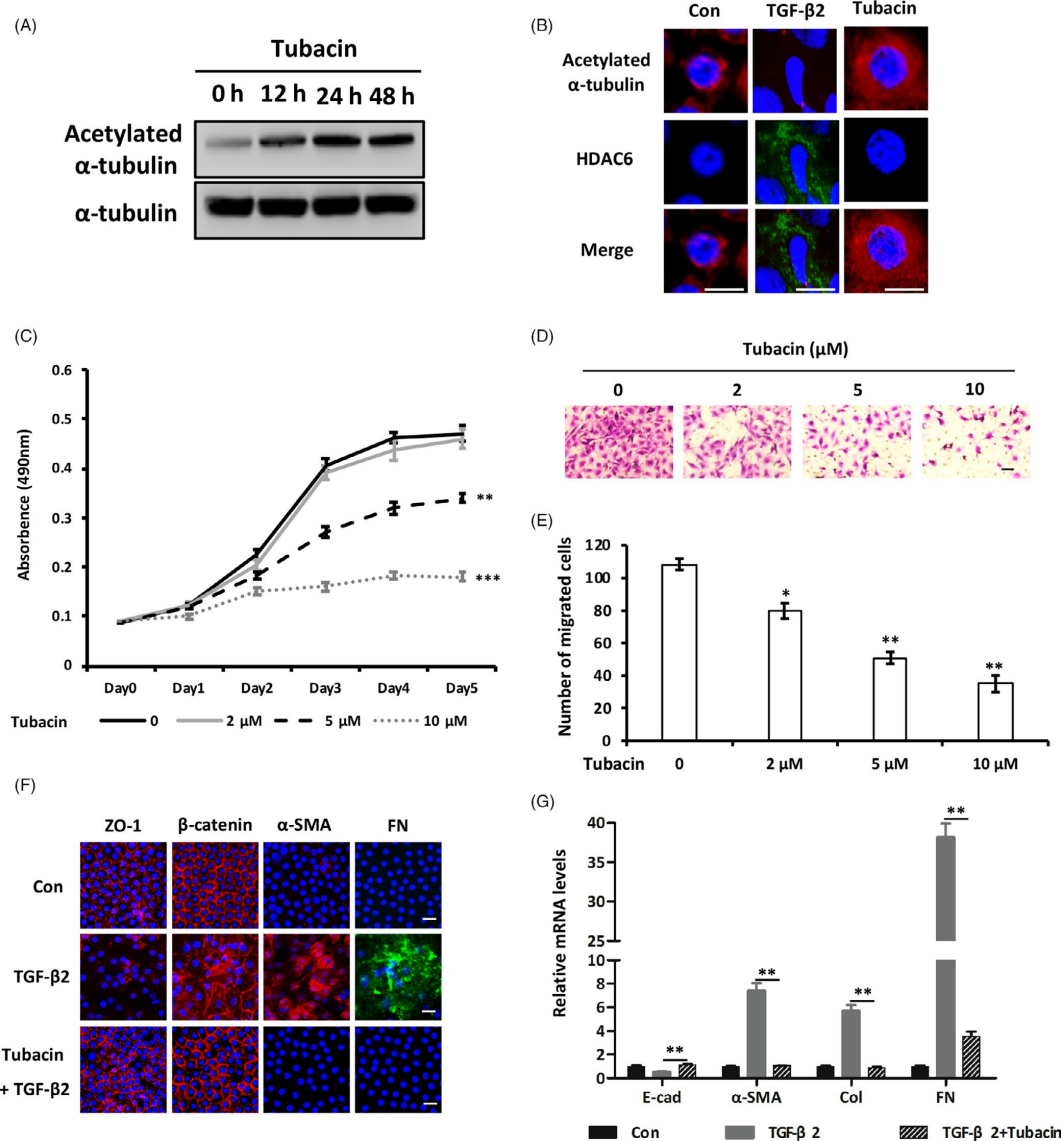
Inhibition of HDAC6 prevents lens fibrosis through α‐tubulin acetylation. A, Western blot of acetylated α‐tubulin levels of lens epithelial explants after Tubacin (5 μmol/L) treatment at different time points (12, 24 and 48). B, Immunofluorescent staining analysis of acetylated α‐tubulin and HDAC6 in lens epithelial explants with TGF‐β2 (5 ng/mL) or Tubacin (5 μmol/L) treatment. C, Proliferative activity of LECs after treatment with different concentrations of Tubacin (0, 2, 5 and 10 μmol/L). D and E, Representative images (D) and quantification (E) of lower chambers following Transwell migration assays under Tubacin‐treated conditions with increased concentrations. F and G, Changes of EMT‐related markers after Tubacin (5 μmol/L) treatment in TGF‐β2 (5 ng/mL) induced fibrosis model of lens epithelial explants detected by immunofluorescent staining (F) and qPCR analysis (G). All scale bars: 20 μm. *P < .05, **P < .01 and ***P < .001 by Student's *t* test, all n = 3 per group. Data are shown as mean ± SD

Based on the mechanistic rationale, we evaluated whether pharmacological targeting of HDAC6 deacetylase with specific inhibitor, Tubacin, could phenocopy the impact of HDAC6 genetic knockdown on lens fibrosis. Tubacin promoted α‐tubulin acetylation significantly in lens epithelial explants (Figure 6A,B). Both proliferation and migration of LECs were inhibited notably as concentration of Tubacin increased (Figure 6C‐E). Meanwhile, Tubacin restored epithelial makers while repressed mesenchymal markers following TGF‐β2 stimulation (Figure 6F,G). Consistent with the role of miR‐22‐3p overexpression and HDAC6 inhibition on lens fibrosis, α‐tubulin acetylation prevented LECs transforming towards mesenchymal‐like cells, indicating (de)acetylated‐α‐tubulin as a mediator of HDAC6 and miR‐22‐3p in the pathogenesis of fibrotic cataract.

### 3.7 α‐tubulin acetylation with Tubacin, an HDAC6 deacetylase inhibitor, prevents TGF‐β2‐induced ASC

**Figure 7:**
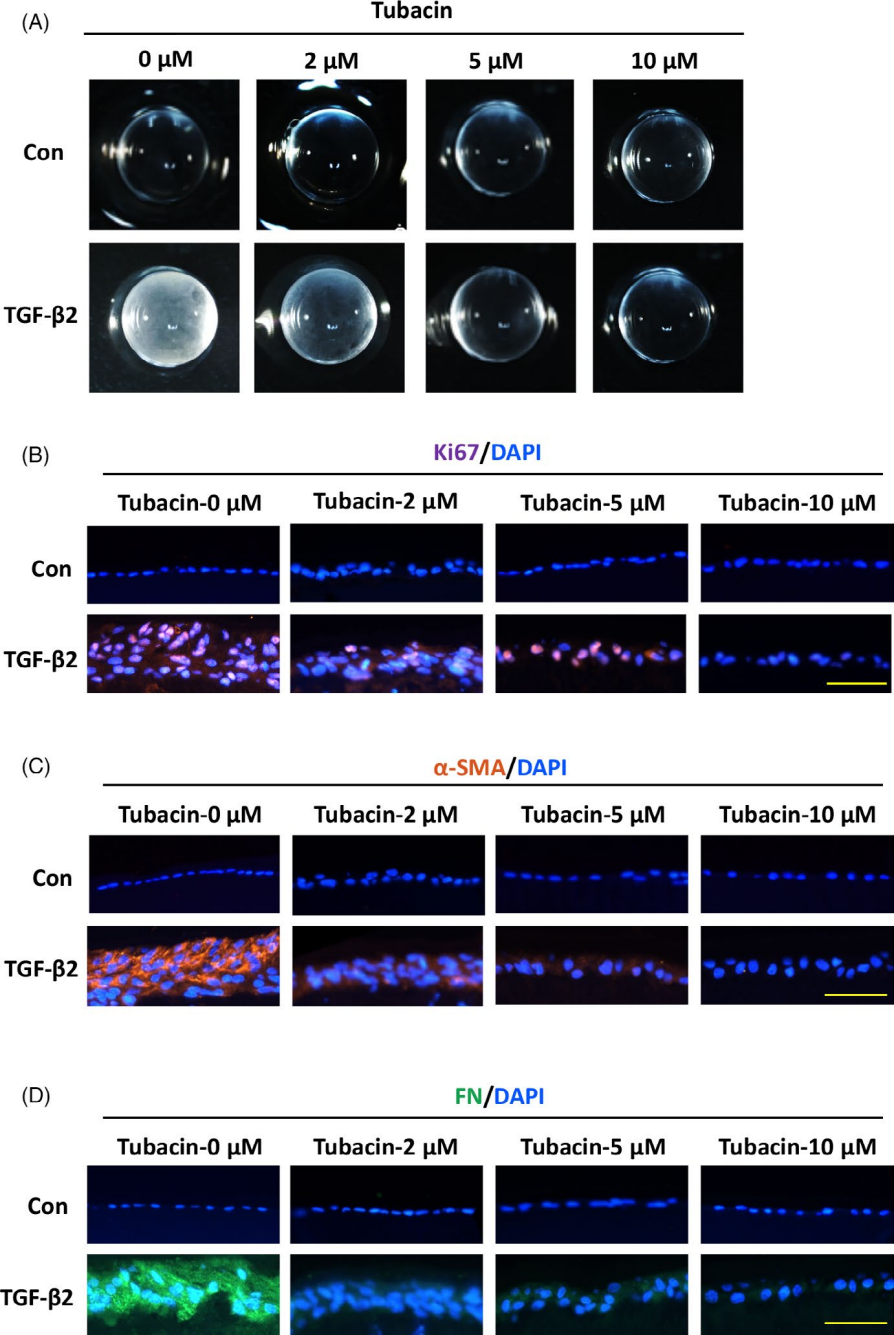
α‐tubulin acetylation with Tubacin, an HDAC6 deacetylase inhibitor, prevents TGF‐β2‐induced ASC. A, Representative images of lenses treated by different concentrations of Tubacin (0, 2, 5 and 10 μmol/L) with or without TGF‐β2 (5 ng/mL) for 5 d. B‐D, Immunofluorescence staining for Ki67 (B), α‐SMA (C) and FN (D) under indicated conditions. All scale bars: 50 μmol/L. *P < .05 by Student's *t* test, all n = 3 per group. Data are shown as mean ± SD

To study whether blockade of HDAC6 deacetylase abrogates lens fibrosis in a more sophisticated system, we delivered different concentrations of Tubacin to the semi‐in vivo ASC model.^30^ Whole rat lenses were cultured with 5 ng/mL of TGF‐β2 for 5 days and developed obvious cloudy opacities beneath the lens capsule (Figure 7A) with disorganized proliferation and aberrant fibrosis evidenced by increased Ki67, α‐SMA and FN (Figure 7B‐D). In contrast, Tubacin reversed TGF‐β2‐induced ASC as concentration increased and the lenses treated with 5 and 10 μmol/L Tubacin remained transparent as normal lens (Figure 7A). Additionally, lenses treated by Tubacin not only retained mono‐layered lens epithelium with decreased proliferation but also maintained epithelial state by preventing mesenchymal cells accumulation and ECM secretion with decreased α‐SMA and FN (Figure 7B‐D). Collectively, these findings reveal that α‐tubulin acetylation via HDAC6 inhibition can suppress ASC formation.

## 4 DISCUSSION

Investigating the mechanism underlying lens fibrosis is important for improving strategies for the prevention and treatment of fibrotic cataract. Here, we show that ‘miR‐22‐3p ‐HDAC6‐α‐tubulin (de)acetylation’ signalling axis plays a pivotal role in hyperproliferation, migration EMT of LECs. MiR‐22‐3p, which is downregulated during PCO and TGF‐β2‐induced EMT, is responsible for the maintenance of epithelial markers and prevention of mesenchymal transition by targeting HDAC6 post‐transcriptionally and increasing α‐tubulin acetylation. Tubacin, a specific inhibitor of HDAC6 deacetylase, could elevate α‐tubulin acetylation and thereby prevent disorganized growth and fibrosis opaque formation in TGF‐β2‐induced ASC model, implying the therapeutic potential of Tubacin for the intervention of fibrotic cataract.

MiR‐22‐3p is one of the most evolutionarily conserved microRNAs in vertebrates, especially in mammary progenitor cells, suggesting that it may be important for the basic physiology functions.^31^ Accumulating evidence has demonstrated that miR‐22‐3p was extensively involved in hyperproliferative diseases, such as cancer and fibrosis. MiR‐22‐3p was downregulated in diverse cancers and had been identified as a tumour suppressor via negative regulation mainly on proliferation and invasion.^32^ Furthermore, miR‐22‐3p also suppressed cell proliferation, migration and EMT in fibrotic diseases such as arteriosclerosis obliterans, post‐myocardial infarction and cardiac fibrosis.^33^, ^34^ Meanwhile, we reported earlier that miR‐22 was downregulated in cataractous human lenses through microarray.^35^ However, whether and how miR‐22‐3p regulates lens fibrosis is still largely unknown. In the current study, we demonstrated that miR‐22‐3p was decreased in both fibrotic plaques from fibrotic cataract patients and under TGF‐β2 stimulation, as well as exerted inhibitory effects on the fibrosis processes through HDAC6.

Previous works also have shown the EMT‐promoting role of HDAC6 by deacetylating α‐tubulin and reorganization of the cytoskeletal architecture in ﻿mammary epithelial cells.^36^ Apart from microtubule α‐tubulin, the roles of other cytoplasmic substrates of HDAC6, including the chaperone Hsp90, cortactin or others to be identified, should also be considered during fibrosis. Studies show that both Hsp90 and cortactin are involved in EMT regulation.^37^, ^38^, ^39^ Increased Hsp70 and Hsp90 are shown to prevent EMT and enhance cell survival of rat lens epithelium_,_ ^40^ and tyrosine phosphorylated cortactin is required for E‐cadherin stability.^41^ Additionally, HDAC6 deacetylates β‐catenin and thus represses its phosphorylation, leading to its nuclear translocation and promoting proliferation of cancer cells.^42^, ^43^ Hence, further studies are still needed to reveal whether other cytoplasmic substrates of HDAC6 are involved in miR‐22‐3p regulated lens fibrosis.

In addition, HDAC6 has functions beyond its deacetylase activity due to the ubiquitin‐binding motif at its C‐terminus by which HDAC6 binds to poly‐ubiquitinated proteins and transports them into autophagosomes through microtubules for clearance by autophagy.^44^ Autophagy is an intracellular homoeostatic process associated with the degradation and recycling of proteins and cellular organelles in response to signals from the microenvironment.^45^ HDAC6‐mediated autophagy is critical for neurodegenerative disorders with aggregated proteins_,_ ^44^ and accumulating studies have identified the involvement of autophagy during fibrosis. For instance, renal fibrosis can be attenuated by reducing autophagy through HDAC6 reduction and microtubule disruption.^46^ Our previous study also revealed the potential role of autophagy on EMT of retinal pigment epithelial cells during retinal fibrotic pathogenesis.^47^ Moreover, miR‐22 has been mentioned in cancer progression and other pathological processes through autophagy.^48^, ^49^, ^50^ Thus, the effect of miR‐22‐3p‐ or HDAC6‐mediated autophagy might also be considered as potential mechanism during lens fibrosis, and further investigation focusing on autophagy will be performed in our future work.

A key feature of EMT is the loss of cell‐cell junctions. More than adhesive functions, E‐cadherin/β‐catenin complex plays a crucial role in controlling EMT whereby loss of E‐cadherin promotes the release of β‐catenin from the cell membrane, thus stabilizing β‐catenin in the cytosol for nuclear import to promote the expression of EMT‐related genes.^51^, ^52^ Moreover, β‐catenin is a key mediator in the Wnt signalling implicated in lens development and cataract formation.^53^, ^54^, ^55^ In this study, both TGF‐β2 treatment and miR‐22‐3p inhibition to lens epithelial explants decreased E‐cadherin expression and promoted β‐catenin translocating into cytoplasm. In contrast, miR‐22‐3p overexpression, HDAC6 inhibition and α‐tubulin acetylation retained β‐catenin to the cell membrane and restored E‐cadherin expression levels, thereby maintaining the lens epithelial state. Additionally, HDAC6 was responsible for Wnt‐ and EGF‐induced nuclear localization of β‐catenin through deacetylating β‐catenin at different lysine sites in cancer cells.^42^, ^43^ Together, these results indicated that miR‐22‐3p and HDAC6 might modulate lens fibrosis through controlling the stability of the E‐cadherin/β‐catenin complex and related pathways. Hence, it would be interesting to explore whether ‘miR‐22‐3p‐HDAC6‐(de)acetylated α‐tubulin’ axis induced by TGF‐β2 modulates lens fibrosis via regulation of the E‐cadherin/β‐catenin complex through crosstalk with Wnt signalling or other pathways.

In summary, we identify miR‐22‐3p as an anti‐fibrotic factor during lens fibrosis. We have established a post‐translational mechanism by which miR‐22‐3p directly targets HDAC6 and thereby promotes α‐tubulin acetylation to control lens fibrosis by maintaining the homoeostasis of the lens epithelium, providing a novel mechanistic insight for prevention and treatment of fibrotic cataract and other fibrotic diseases.

## CONFLICT OF INTEREST

The authors declare that they have no conflict of interest.

## AUTHOR CONTRIBUTIONS

W.X, W.L and S.Y performed the experiments. C.B, X.L, C.J and H.M analysed the data. T.X, and W.J designed the project. H.S, Z.Y and L.Y designed the project and wrote the manuscript.

## Supporting information

Table S1

## Data Availability

All data generated or analysed during this study are included in this article.
